# Incidence, aetiology and related comorbidities of cirrhosis: a Swedish population-based cohort study

**DOI:** 10.1186/s12876-020-01239-6

**Published:** 2020-04-03

**Authors:** Juan Vaz, Berne Eriksson, Ulf Strömberg, David Buchebner, Patrik Midlöv

**Affiliations:** 1 Department of Clinical Sciences in Malmö, Center for Primary Health Care Research, Lund University, Malmö, Sweden; 2 Department of Internal Medicine, Halland Hospital Halmstad, Halmstad, Sweden; 3 Krefting Research Centre, Institute of Medicine, University of Gothenburg, Gothenburg, Sweden; 4 Department of Research and Development, Region Halland, Halmstad, Sweden; 5 Health Metrics Unit, Institute of Medicine, Sahlgrenska Academy at University of Gothenburg, Gothenburg, Sweden

**Keywords:** Cirrhosis, Incidence, Aetiology, Comorbidities, Sweden

## Abstract

**Background:**

The incidence of cirrhosis for individuals in Sweden has previously been reported as stable/low among European countries. However, Swedish population-based studies are scarce and none of them included data from the most recent decade (2010–2019). We aimed to describe the incidence and aetiology of cirrhosis in the Halland region from 2011 to 2018, and to describe the severity and prevalence of liver-related complications and other primary comorbidities at the time of cirrhosis diagnosis.

**Methods:**

We conducted a retrospective cohort study of all patients with cirrhosis in Halland, which has a population of 310,000 inhabitants. Medical records and histopathology registries were reviewed.

**Results:**

A total of 598 patients with cirrhosis were identified. The age-standardised incidence was estimated at 23.2 per 100,000 person-years (95% CI 21.3–25.1), 30.5 (95% CI 27.5–33.8) for men and 16.4 (95% CI 14.3–18.7) for women. When stratified by age, the highest incidence rates were registered at age 60–69 years. Men had a higher incidence rate for most age groups when compared to women. The most common aetiology was alcohol (50.5%), followed by cryptogenic cirrhosis (14.5%), hepatitis C (13.4%), and non-alcoholic fatty liver disease (5.7%). Most patients had at least one liver-related complication at diagnosis (68%). The most common comorbidities at diagnosis were arterial hypertension (33%), type 2 diabetes (29%) and obesity (24%).

**Conclusions:**

Based on previous Swedish studies, our results indicate that the incidence of cirrhosis in Sweden might be considerably higher than previously reported. It is uncertain if the incidence of cirrhosis has previously been underestimated or if an actual increment has occurred during the course of the most recent decade. The increased incidence rates of cirrhosis reported in Halland are multifactorial and most likely related to higher incidence rates among the elderly. Pre-obesity and obesity are common in cirrhosis and non-alcoholic fatty liver disease has become an important cause of cirrhosis in Halland.

## Background

Liver cirrhosis is the end-stage of several chronic liver diseases and accounts for more than one million deaths each year worldwide [1]. It is also the main risk factor for hepatocellular carcinoma (HCC) [1, 2]. Alcohol and non-alcoholic fatty liver disease (NAFLD) are the main causes of cirrhosis in Western and industrialised nations [1].

In Europe, alcohol, hepatitis B, hepatitis C (HCV), and obesity are the main causes of cirrhosis [3]. Sweden has a stable/low incidence of cirrhosis among European nations [3]. In Gothenburg, the second-largest city in Sweden, the crude annual incidence between 1994 and 2003 was estimated at 15.3 per 100,000 [4]. In southern Sweden (Scania County) an incidence of 14.1 per 100,000, between 2001 and 2011, has been reported [5]. In Sweden, cirrhosis occurs mainly due to alcohol-related liver disease and HCV [4, 5]. The overall incidence of HCV in Sweden has slowly decreased since 2014 (15.8 per 100,000, year 2018) [6]. Simultaneously, the prevalence of obesity and type 2 diabetes has been increasing in Sweden during the last decades [7]. In a Swedish population-based study, the authors found non-alcoholic steatohepatitis (NASH) in 4% of all cases of cirrhosis between 2001 and 2011, which was higher than previously reported in Danish, Norwegian and British cohorts [5, 8–10]. To date, it is unclear how the increasing prevalence of obesity and type 2 diabetes has affected the incidence of cirrhosis in Sweden. A major challenge regarding epidemiological studies of cirrhosis in Sweden is the lack of a nationwide cirrhosis registry.

The aim of this retrospective study was to describe the incidence and aetiology of cirrhosis in a well-defined Swedish population at the time of cirrhosis diagnosis, and to describe the severity and prevalence of liver-related complications and other primary comorbidities.

## Methods

### Study population, patient data and definitions

Halland is a county located on the western coast of Sweden. With a stable population (310,665 inhabitants, year 2014), Halland is served by two midsized hospitals, and a smaller one, together referred to as the Halland Hospital.

A search was performed for all patients with cirrhosis diagnosed at Halland Hospital between January 1st 2011 and December 31st 2018. A wide array of cirrhosis-related International Classification of Diseases 10th Revision (ICD-10) codes were searched (Supplementary Table). Further patients were retrieved from the pathology registry using the SNOMED codes T-56 (liver), M-495 (cirrhosis) and M-817 (HCC).

The diagnosis of cirrhosis was established histologically or based on clinical and laboratory findings, combined with characteristic radiological features, such as irregular or nodular liver surface, blunt edges or segmental hypertrophy/atrophy and signs of portal hypertension [11]. We excluded patients with apparent cirrhosis on the basis of clinical and laboratory findings, lacking characteristic radiological features or histology confirming the diagnosis. In addition, we also excluded all patients who were not residents in Halland at diagnosis, or diagnosed under age 18 years, or before January 1st 2011.

Electronic medical records were available from January 1st 2011 at all three participating hospitals. All medical records were reviewed and the following information was obtained: date of birth, sex, weight and length, date of diagnosis, aetiology, diagnostic work-up, complications and comorbidities at diagnosis, use of warfarin, and laboratory results.

A complication was regarded as prevalent if registered during the diagnostic work-up. HCC cases diagnosed during the first 6 months of follow-up were also considered a prevalent complication. All other complications discovered during the follow-up were considered incidental and thereby not reviewed in this manuscript. All registered comorbidities were included if diagnosed for up to 20 years prior to cirrhosis diagnosis.

The body mass index (BMI) was calculated and the patients were graded after their BMI values (kg/m^2^) according to the International Classification of adult underweight (BMI < 18.5), normal weight (BMI 18.5–24.9), pre-obesity (BMI 25.0–29.9), and obesity (BMI > 29.9) defined by WHO [12]. When profuse ascites and/or oedema were observed, the calculation of BMI was performed using the weight recorded after received medical treatment (abdominal paracentesis and/or diuretics).

Ascites was registered when detected clinically and/or in radiological examination. If oesophageal varices were found in gastroscopy, radiology or autopsy, they were registered regardless of size. Variceal bleeding was assumed upon manifest signs of bleeding according to the Baveno IV classification of significant bleeding [13]. Spontaneous bacterial peritonitis was defined as positive culture of ascites or polymorphonuclear leucocyte count > 0.25*10^9^/L. Hepatic encephalopathy was registered if described by the treating physician.

The following aetiological groups were defined: alcohol, HCV, NAFLD, cryptogenic cirrhosis, primary biliary cholangitis, autoimmune hepatitis, and “Other causes”. All recorded causes of cirrhosis were registered and patients were regarded as having alcohol-cirrhosis when a history of long-lasting alcohol abuse was noticed in the medical records. Elevated concentration of phosphatidylethanol (PEth), or (in some cases) carbohydrate-deficient transferrin (CDT), further reinforced the diagnosis. Patients were regarded as having HCV only if diagnosed by an infectious disease specialist. Patients with concomitant alcohol-abuse and HCV were only included in the HCV group. Patients were classified as having NAFLD when the clinicians set out this diagnosis or when substantial evidence for NAFLD was found during the retrospective review of medical charts in patients with previously undefined (cryptogenic) cirrhosis or by biopsy. The NAFLD fibrosis score (NFS) was calculated for all NAFLD patients without a history of biopsy (cut-off: > 0.675 for advanced fibrosis likely) [14, 15]. When the cause of cirrhosis could not be established, the patients were regarded as having cryptogenic cirrhosis. “Other causes” was reserved for specific aetiologies with probable low incidence rate in our cohort, such as primary sclerosing cholangitis and hemochromatosis.

Fibrosis-4 index for liver fibrosis (FIB-4) was calculated as described before (cut-off: > 2.67 for advanced fibrosis likely) [15, 16]. In order to estimate the severity of liver cirrhosis at diagnosis, Model for End-stage Liver Disease (MELD) score, Child-Pugh class, and the Baveno IV stages of cirrhosis were calculated [13, 17]. MELD scores and Child-Pugh class were not calculated for patients with advanced chronic kidney disease or under warfarin-treatment.

### Statistical analysis

Data were expressed as medians and percentiles for continuous variables or as numbers and percentages for categorical ones. Fisher’s test was used for dichotomous variables and chi-square test was performed for non-ordered categorical variables. Missing data (expressed as percentages) were registered for BMI, MELD-score and Child-Pugh class.

Data regarding the population in Halland for the study period (2011–2018) were retrieved from Statistics Sweden (www.scb.se). The crude incidence of cirrhosis was calculated per 100,000 person-years and stratified by year of diagnosis, sex and 5-year age group. Age-standardised incidence rates (ASIR) were also calculated, using the Revised European standard population from 2013 (2013 ESP) [18]. In order to allow comparison with previous Swedish studies, we also reported the ASIR based on the European standard population from 1976 (1976 ESP) [18]. Confidence intervals (CI) of crude incidence rates and ASIR were calculated assuming a Poisson-distributed number of observed cases of cirrhosis.

All tests were two-tailed and conducted at the 5% significance level, using IBM SPSS Statistics for Macintosh (version 25.0, IBM Statistics, Amorak, NY, USA).

## Results

### Study cohort

**Fig. 1:**
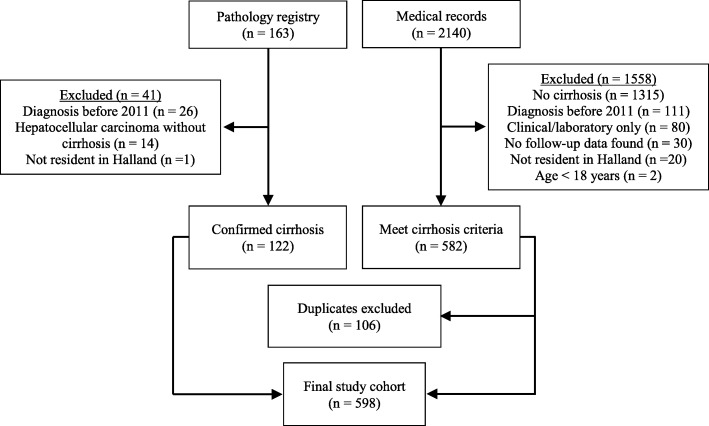
Cirrhosis in Halland (Sweden), 2011–2018. Identification Flowchart

Through the pathology registry, a total of 163 patients were identified and their clinical charts were reviewed. Through medical records, a second group consisting of 2140 patients were identified, and their clinical charts were studied. The final study cohort consisted of 598 patients (Fig. 1).

Radiological examinations, combined with clinical signs and laboratory findings, were responsible for the diagnoses of 436 patients (72.9%). Biopsy and/or autopsy accounted for the diagnosis in 162 patients (27.1%). The FIB-4 was calculated for 545 patients (91.1%). Among patients without biopsy-verified cirrhosis, the median FIB-4 was 4.99 (calculated for 92.2% of these patients). Sixteen patients (2.67%), without known liver disease before death, were first diagnosed at autopsy. The cause of cirrhosis in those cases was retrieved either via biopsies or through medical records.

Two or more possible aetiologies were registered in 79 patients (13.2%). Alcohol was the most common aetiology, 302 patients (50.5%), followed by cryptogenic cirrhosis, 87 patients (14.5%). A total of 80 patients had HCV (13.4), and 50 (62.5%) of them had alcohol-abuse as a contributing cause of cirrhosis. NAFLD was found in 34 patients (5.7%) and was diagnosed by biopsy in 21 of those patients (61.8%). The median NFS among those without biopsy was 3.92. Cirrhotic primary sclerosing cholangitis was only found in 10 patients (1.7%), which were included in the aetiological group “Other causes”. Hepatitis B was found in seven patients, alpha-1-antitrypsin deficiency in four patients, hemochromatosis in three patients, and Budd-Chiari syndrome in two patients. Other uncommon causes, such as congenital diseases and porphyria (eight cases), were also included in “Other causes” (Table 1).

**Table 1:** Baseline characteristics at the time of diagnosis of cirrhosis in Halland, 2011–2018

	Alcohol	HCV	Cryptogenic	NAFLD	PBC	AIH	Other causes	Overall
Overall, n (%)	302 (50.5)	80 (13.4)	87 (14.5)	34 (5.7)	31 (5.2)	30 (5.0)	34 (5.7)	598 (100)
Male, n (%)	212 (70)	55 (69)	59 (68)	19 (56)	5 (16)	6 (20)	24 (71)	380 (64)
Female, n (%)	90 (30)	25 (31)	28 (32)	15 (44)	26 (84)	24 (80)	10 (29)	218 (36)
Median age (years) (10–90 percentile)	65 (52–76)	57 (46–67)	76 (63–88)	75 (62–86)	72 (58–81)	69 (46–85)	64 (30–83)	66 (50–81)
**Comorbidities, n (%)**								
Hypertension	96 (32)	17 (21)	32 (37)	23 (68)	12 (39)	7 (23)	9 (27)	196 (33)
Ischaemic heart disease	59 (20)	4 (5)	27 (31)	11 (32)	7 (23)	2 (6.7)	4 (12)	114 (19)
Chronic heart failure	37 (12)	2 (2.5)	35 (40)	8 (24)	1 (3.2)	0 (0)	3 (8.8)	86 (14)
Type 2 diabetes	78 (26)	6 (21)	37 (43)	18 (53)	9 (29)	4 (13)	8 (24)	171 (29)
Obesity ^a^	81 (27)	18 (23)	10 (11)	24 (70)	4 (13)	5 (17)	1 (2.9)	143 (24)
**Severity, n (%)**^b^								
MELD (median)	14	9	11.5	9	6	10	10	11
MELD < 10	70 (23)	49 (61)	30 (34)	16 (47)	23 (74)	14 (47)	14 (41)	216 (36)
MELD 10–15	84 (28)	11 (15)	19 (22)	6 (18)	4 (13)	6 (20)	11 (32)	142 (24)
MELD ≥15	140 (46)	18 (23)	27 (31)	9 (26)	1 (3.2)	9 (30)	6 (18)	210 (35)
Child-Pugh A	80 (26)	48 (60)	19 (22)	16 (47)	24 (77)	15 (50)	16 (47)	217 (36)
Child-Pugh B	122 (40)	21 (26)	47 (54)	12 (35)	4 (13)	10 (33)	12 (35)	228 (38)
Child-Pugh C	91 (30)	11 (15)	10 (11)	3 (8.8)	0 (0)	4 (13)	3 (8.8)	122 (20)
Baveno IV, 1–2	121 (40)	53 (66)	33 (38)	25 (74)	28 (90)	22 (73)	23 (68)	305 (51)
Baveno IV, 3–4	181 (60)	27 (34)	54 (62)	9 (26)	3 (10)	8 (27)	11 (32)	292 (49)
**Complications, n (%)**								
Any complication	227 (75)	44 (55)	74 (85)	23 (68)	7 (21)	15 (50)	19 (56)	409 (68)
Ascites	175 (58)	24 (30)	52 (60)	8 (24)	3 (10)	8 (27)	10 (29)	280 (47)
Oesophageal varices	119 (39)	26 (33)	31 (36)	13 (38)	4 (13)	9 (30)	13 (38)	215 (36)
Variceal bleeding	19 (6.3)	4 (5)	6 (6.9)	1 (2.9)	0 (0)	1 (3.3)	1 (2.9)	32 (5.4)
Portal vein thrombosis	10 (3.3)	7 (8.8)	10 (11)	0 (0)	0 (0)	0 (0)	5 (15)	32 (5.4)
Encephalopathy	34 (11)	5 (5)	2 (2.3)	1 (2.9)	0 (0)	2 (6.7)	0 (0)	43 (7.2)
Spontaneous bacterial peritonitis	5 (1.7)	1 (1.3)	0 (0)	0 (0)	1 (3.2)	0 (0)	1 (2.9)	8 (1.3)
Hepatocellular carcinoma	21 (6.9)	16 (20)	29 (33)	5 (15)	1 (3.2)	1 (3.3)	2 (5.9)	75 (13)

*AIH* Autoimmune Hepatitis, *HCV* Hepatitis C, *MELD* Model for End-Stage Liver Disease, *NAFLD* Non-alcoholic Fatty Liver Disease, *PBC* Primary Biliary Cholangitis

^a^Obesity defined as body mass index > 30 kg/m^2^

^b^MELD-score and Child Pugh-score calculated for 95% of patients

The median age at diagnosis was 66 years and most patients were male (63.5%). Among most aetiologies, a male predominance was registered (Table 1). As expected, most patients with PBC and AIH were female (84 and 80% respectively). Cryptogenic cirrhosis and NAFLD were associated with the oldest patients. The BMI-values were calculated for 96.8% of the patients. Most patients, 352 (58.9%) were pre-obese or obese at diagnosis, and only 23 patients (3.8%) were underweight (data not shown). The highest BMI-values were registered in NAFLD patients, which also had the highest prevalence of obesity (70%). The most common cardiovascular comorbidities were the following: arterial hypertension (32.8%), ischaemic heart disease (19.1%), and chronic heart failure (14.4%). Type 2 diabetes was registered in 171 patients (28.6%). Cardiovascular disease and diabetes were frequently found in NAFLD and cryptogenic cirrhosis (Table 1).

The MELD scores were calculated for 94.9% of the cohort and the median MELD score was 11 (Table 1). Child-Pugh scores were calculated for 94.8% of the cohort. Patients were stratified into three MELD-groups: MELD < 10, MELD 10–15, and MELD ≥15. PBC had the lowest median MELD, and 90% of the patients had compensated disease at diagnosis (Table 1). MELD ≥15 and/or Child-Pugh C were more common in alcohol-cirrhosis. Decompensation (Baveno IV 3–4) was often seen in cryptogenic cirrhosis (62%) and alcohol (60%).

### Incidence

Sixteen patients were first diagnosed at autopsy. Common for these patients was the lack of a medical history for liver disease during their lifetime. However, in most autopsy reports (15 cases), cirrhosis was identified as a relevant factor for the cause of death. Cirrhosis was stated as the final cause of death for one patient. Thus, as the clinical relevance of cirrhosis in all patients with “silent” liver disease can be assumed hence justifying that these patients are included. Nevertheless, case-restricted incidence rates are also presented (Table 2). The overall crude annual incidence of cirrhosis, for the period 2011–2018, was estimated at 23.8 per 100,000 (95% CI 21.9–25.8), 30.3 for men (95% CI 27.3–33.5) and 17.3 for women (95% CI 15.1–19.8). The overall crude annual incidence of cirrhosis in adults (18 years and older), for the period 2011–2018, was estimated at 30.2 per 100,000 (95% CI 27.8–32.7), 38.8 for men (95% CI 35.0–42.9) and 21.8 for women (95% CI 19.0–24.9). The overall ASIR calculated according to 2013 ESP was 23.2 per 100,000 person-years (95% CI 21.3–25.1); 30.5 (95% CI 27.5–33.8) for men and 16.4 (95% CI 14.3–18.7) for women. The overall ASIR calculated according to 1976 ESP was 17.1 per 100,000 person-years (95% CI 15.7–18.6); 22.6 (95% CI 20.3–25.1) for men and 11.9 (95% CI 10.2–13.6) for women (Table 2).

**Table 2:** Incidence rates per 100,000 person-years among patients diagnosed with cirrhosis in Halland, 2011–2018

	Overall	Male	Female
Annual crude incidence (95% CI)	23.8 (21.9–25.8)	30.3 (27.3–33.5)	17.3 (15.1–19.8)
Case-restricted annual crude incidence (95% CI)	23.2 (21.3–25.1)	29.3 (26.4–32.5)	17.0 (14.8–19.4)
Annual crude incidence among adults (95% CI)	30.2 (27.8–32.7)	38.8 (35.0–42.9)	21.8 (19.0–24.9)
Case-restricted annual crude incidence among adults (95% CI)	29.4 (27.1–31.9)	37.6 (33.8–41.6)	21.4 (18.6–24.5)
ASIR, 2013 ESP (95% CI)	23.2 (21.3–25.1)	30.5 (27.5–33.8)	16.4 (14.3–18.7)
Case-restricted ASIR, 2013 ESP (95% CI)	22.5 (20.7–24.5)	29.5 (26.5–32.7)	15.9 (13.8–18.2)
ASIR, 1976 ESP (95% CI)	17.1 (15.7–18.6)	22.6 (20.3–25.1)	11.9 (10.2–13.6)
Case-restricted ASIR, 1976 ESP (95% CI)	16.9 (15.3–18.2)	21.9 (19.7–24.4)	11.6 (10.0–13.4)

*ASIR* Age-standardized incidence rate, *CI* Confidence Interval, *ESP* European Standard Population. Adult: age > 18 years old. Case-restricted: 16 patients excluded due to the lack of previous history of liver disease during lifetime but cirrhosis found at autopsy

**Fig. 2:**
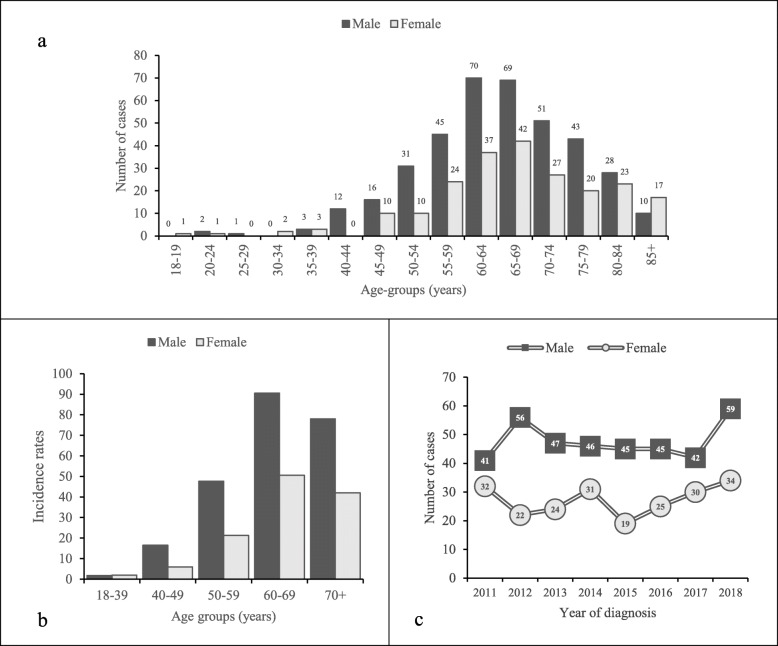
A cohort comprising 598 patients with liver cirrhosis in Halland (Sweden), 2011–2018. **a** Number of cases stratified by sex and age-groups. **b** Incidence rates 2011–2018, per 100,000 person-years, stratified by sex and age-groups. **c** Number of cases stratified by sex and year of diagnosis

The highest incidence rates were registered at age 60–69 years, when stratified by age (Fig. 2). This trend was observed both for men and women. Moreover, men had a higher incidence rate for most age groups when compared to women (Fig. 2).

### Complications

The most observed complications at diagnosis were: ascites (47%), oesophageal varices (36%), and HCC (12.5%). A total of 409 patients (68%) had at least one complication at diagnosis (Table 1). Ascites was mostly found in cryptogenic cirrhosis and in alcohol-cirrhosis. Prevalent HCC at diagnosis was more frequent in cryptogenic cirrhosis (33%) and HCV (20%). HCC was also relatively frequent in NAFLD (15%).

## Discussion

The estimated crude incidence rate in our cohort was more than 68% higher (23.8 vs 14.1, per 100,000) than previously reported in a Swedish cohort from Scania [5]. In order to allow comparison, we have also calculated the ASIR according to the 1976 ESP. Even here, we found higher incidence rates when compared to the study from Scania: 22.6 vs 17.8 per 100,000 person-years for men, and 11.9 vs 8.8 per 100,000 person-years for women [5]. When calculated according to the 2013 ESP, the ASIR were even higher in our cohort (Table 2). The 2013 ESP calculated ASIR were also very similar to the crude annual incidence rates (Table 2). A comparison between the 1976 ESP and the 2013 ESP has shown that the latter is more representative for old and very old individuals (75+ years) and has no major impact on incidence rates estimated for younger individuals [19].

Our cohort was comprised of 140 patients (23.4%) who were 75+ years old at diagnosis. This might, in part, account for the higher median age reported by us compared to earlier Swedish studies (66 years vs 60 years) [4, 5]. An important factor affecting the median age in our cohort is the higher age at diagnosis among patients with alcohol-related cirrhosis (65 years vs 61 years) [5]. Delay in seeking medical attention might be responsible for this situation; most patients with alcohol-cirrhosis were diagnosed first during an episode of severe acute alcohol hepatitis, alcohol withdrawal syndrome, or anaemia with patent gastrointestinal bleeding (data not shown).

The mean annual alcohol consumption per capita between 2002 and 2016 in Halland was estimated to be higher than the ones registered in Scania and in Gothenburg: 10.8 L, 9.2 L and 10.2 L, respectively [20]. We reported 50.5% alcohol-related cirrhosis, which does not meaningfully differ from studies from Gothenburg and the Scania region (50 and 49%) [4, 5]. Thus, while the higher alcohol-consumption in Halland is an important factor, this does not appear to be the sole explanation concerning the differences in reported incidence rates.

Only 4% of all cirrhosis was NASH-related in Scania [5]. Since BMI values were not available, the authors speculated that some cases with cryptogenic cirrhosis could have been unrecognised NASH. There is no distinction done between NASH and non-alcoholic fatty liver (NAFL) in our study. There is increasing evidence indicating that fibrosis progression can also occur in NAFL [15, 21]. Fibrosis stage, independent of NASH occurrence or not at diagnosis, is the most important predictive factor for mortality and time to development of severe liver disease in biopsy-proven NAFLD [22]. NAFLD cirrhosis was found in 5.7% of our cohort, which to date is the highest rate reported in a Swedish study. Moreover, a significant amount of our patients were classified as cryptogenic cirrhosis (14.5%). It has previously been shown that NAFLD can lose typical histological features during the increasing stages of fibrosis thus resulting in the diagnosis of cryptogenic cirrhosis instead [23]. Thus, even if the two might be different entities [24], NAFLD-associated cirrhosis is frequently misdiagnosed as cryptogenic cirrhosis [25]. If this was the case, NAFLD could have been the second most common cause of cirrhosis in our cohort.

HCV is less common in our cohort (13.4%) when compared to Scania and Gothenburg (21.6 and 21.2% respectively) [4, 5]. Since the incidence of HCV has only decreased slightly in Sweden during the last decades, the differences in HCV cirrhosis might rather be related to the local prevalence of persons who inject drugs, which might differ between the three regions [6].

Another important difference is the high prevalence of HCC at diagnosis in our cohort when compared to Scania (12.5% vs 5.7%) [26]. We have observed that 47% of these patients were 75+ years and received care mainly via surgical departments. A major part of HCC patients with concomitant cirrhosis lacked proper ICD-10 codes for the latter (data not shown). These results are in-line with reports from the USA [27, 28] The causes of the elevated prevalence of HCC at diagnosis in Halland are beyond the scope of this manuscript but will be the subject for future investigations.

Some limitations must be considered, most of them related to the retrospective nature of our study. As neither ultrasound nor CT have perfect diagnostic sensitivity and specificity, it can be assumed that a minor quantity of cases have been under- or over-diagnosed [11]. However, we have excluded 80 patients without radiological or histological data supporting the diagnosis. It is then possible that some patients with cirrhosis were erroneously excluded. Patients with prior long-lasting alcohol-abuse might have been misclassified when alcohol-abuse was denied by the patients.

Some cases of cryptogenic cirrhosis might have wrongly been classified as NAFLD and vice versa. Indeed, we suspect that a major part of the patients classified as cryptogenic cirrhosis had NAFLD instead. Similarities regarding the age at diagnosis, the prevalence of ischaemic cardiac disease and type 2 diabetes between the two groups are striking.

In Fig. 2, an increment in the cases of cirrhosis during 2018, mainly among men, can be observed. This increment is partially explained by a higher incidence of “silent” cirrhosis at autopsy as during 2018 a total of 8 patients (7 male) were diagnosed by this means.

A strength in our study was the high availability of reliable patient data. Halland has a single-payer healthcare system, centralised management of patients with liver disease and universal computerised medical records; including histopathology and autopsy registries. Halland Hospital and most primary care centres share the same medical record system, which allowed a comprehensive review of medical charts. All histopathological studies and autopsies are centralised to a single centre. The Dept. of Infectious Diseases is responsible for the follow-up of all known HCV patients in the county. Additionally, scanned medical records, including laboratory findings, between 2000 and 2011 were also available for most patients.

Due to its geographical proximity, a similar epidemiological database regarding cirrhosis should be registered in Halland, Gothenburg and Scania. Nevertheless, it must be emphasised that Halland, like Scania, is a county, meaning that incidence rates or aetiology of cirrhosis in Halland can be entirely, moderately, or non-representative for the general Swedish population.

## Conclusions

The increased incidence rates of liver cirrhosis reported in Halland 2011–2018 are multifactorial and most likely related to higher incidence rates among the elderly, which might often have unrecognised, undefined (cryptogenic) cirrhosis and HCC at diagnosis. These patients have also a higher prevalence of liver-related complications at diagnosis.

While the incidence of HCV is slightly decreasing in Sweden, obesity and type 2 diabetes are becoming more common. Pre-obesity and obesity are common in cirrhosis and NAFLD has become an important cause of cirrhosis in Halland. Cardiovascular comorbidities, diabetes and obesity are common in NAFLD at the time of diagnosis.

Based on previous Swedish studies, our results indicate that the incidence of cirrhosis in Sweden might be considerably higher than previously reported. It is uncertain if the incidence of cirrhosis has prevously been underestimated or if an actual increment has occurred during the course of the most recent decade. The establishment of a nationwide liver cirrhosis registry in Sweden is highly needed.

## Supplementary information

**Additional file 1: Table S1**. Diagnoses (ICD-10) used to identify possible patients with cirrhosis in Halland (Sweden), 2011–2018.

## Data Availability

The datasets generated and/or analysed during the current study are not publicly available due to legal and ethical restrictions but anonymised datasets are available from the corresponding author on reasonable request.

## Abbreviations

ASIR: Age-standardised incidence rate
BMI: Body mass index
CI: Confidence intervals
ESP: European standard population
FIB-4: Fibrosis-4 index
HCC: Hepatocellular carcinoma
HCV: Hepatitis C
NAFL: Non-alcoholic fatty liver
NAFLD: Non-alcoholic fatty liver disease
NASH: Non-alcoholic steatohepatitis
NFS: NAFLD fibrosis score
MELD: Model for End-stage Liver Disease
