# Supplementary material for: Incidence, aetiology and related comorbidities of cirrhosis: a Swedish population-based cohort study
Source: BMC Gastroenterol. 2020 Apr 3;20:84. doi: 10.1186/s12876-020-01239-6 (PMC7118963; doi:10.1186/s12876-020-01239-6)
Supplement: Supplementary file 1 — Additional file 1: Table S1. Diagnoses (ICD-10) used to identify possible patients with cirrhosis in Halland (Sweden), 2011–2018. [file 12876_2020_1239_MOESM1_ESM.docx]

| Supplementary Table. Diagnoses (ICD-10) used to identify possible patients with cirrhosis in Halland (Sweden), 2011-2018 | |
| --- | --- |
| Chronic viral hepatitis | |
| B18.0 | Chronic viral hepatitis B with delta-agent |
| B18.1 | Chronic viral hepatitis B without delta-agent |
| B18.2 | Chronic viral hepatitis C |
| B18.8 | Other chronic viral hepatitis |
| B18.9 | Chronic viral hepatitis, unspecified |
| Malignant neoplasm of liver and intrahepatic bile ducts | |
| C22.0 | Liver cell carcinoma |
| C22.1 | Intrahepatic bile duct carcinoma |
| C22.7 | Other specified carcinomas of liver |
| C22.8 | Malignant neoplasm of liver, primary, unspecified as to type |
| C22.9 | Malignant neoplasm of liver, not specified as primary or secondary |
| Alcoholic liver disease | |
| K70.1 | Alcoholic hepatitis |
| K70.2 | Alcoholic fibrosis and sclerosis of liver |
| K70.3 | Alcoholic cirrhosis of liver |
| K70.4 | Alcoholic hepatic failure |
| K70.9 | Alcoholic liver disease, unspecified |
| Chronic hepatitis, not elsewhere classified | |
| K73.0 | Chronic persistent hepatitis, not elsewhere classified |
| K73.2 | Chronic active hepatitis, not elsewhere classified |
| K73.8 | Other chronic hepatitis, not elsewhere classified |
| K73.9 | Chronic hepatitis, unspecified |
| Fibrosis and cirrhosis of liver | |
| K71.7 | Toxic liver disease with fibrosis and cirrhosis of liver |
| K74.3 | Primary biliary cirrhosis |
| K74.4 | Secondary biliary cirrhosis |
| K74.5 | Biliary cirrhosis, unspecified |
| K74.6 | Other and unspecified cirrhosis of the liver |
| Miscellaneous codes | |
| K72.1 | Chronic hepatic failure |
| K72.9 | Hepatic failure, unspecified |
| K75.4 | Autoimmune hepatitis |
| K76.0 | Fatty (change of) liver, not elsewhere classified |
| K76.5 | Hepatic veno-occlusive disease |
| K83.0 | Cholangitis (including primary sclerosing cholangitis) |
| Z94.4 | Liver transplant status |
| Complications associated to liver cirrhosis | |
| K76.6 | Portal hypertension |
| K76.7 | Hepatorenal syndrome |
| I85.0 | Esophageal varices |
| ICD-10: International Classification of Diseases, 10^th^ Revision | |
